# Correlation between cerebellar metabolism and post-stroke depression in patients with ischemic stroke

**DOI:** 10.18632/oncotarget.21063

**Published:** 2017-09-19

**Authors:** Lei Zhang, Muzi Li, Rubo Sui

**Affiliations:** ^1^ School of Nursing, Jinzhou Medical University, Jinzhou, Liaoning, China; ^2^ Department of Neurology, First Affiliated Hospital of Jinzhou Medical University, Jinzhou, Liaoning, China

**Keywords:** post-stroke depression, cerebellum, metabolism, magnetic resonance spectroscopy

## Abstract

The neurochemical changes that occur in the brain of patients with post-stroke depression (PSD) are not fully understood. This study aims to explore the correlation between cerebellar metabolism changes and PSD using proton magnetic resonance spectroscopy (^1^H-MRS). Participants were assigned to 3 groups: 60 patients with PSD (PSD group), 60 stroke patients without depression (NOPSD group), and 60 healthy volunteers (HEAL group). T1 WI, T2 WI, DWI and ^1^H-MRS examination were performed for patients at 14 days, 3 months after the stroke, respectively, and for healthy volunteers once when included in the study. Cho/Cr and Cho/NAA ratios in the cerebellar hemisphere contralateral to the lesion were higher in the PSD group than those in the HEAL and NOPSD groups on 14th day after the stroke (*P* < 0.05). In PSD group, Cho/Cr and Cho/NAA ratios in the cerebellar hemisphere contralateral to the lesion were positively correlated to the HAMD scale scores at both 14 days and 3 months after stroke (*P* < 0.05); Higher Cho/Cr and Cho/NAA ratios, and lower NAA/Cr ratio in the cerebellar hemisphere contralateral to the lesion were observed at 3 months after stroke compared to that at 14 days after stroke. Cerebellar damage may lead to PSD, and the degree of cerebellar damage may be associated with severity of PSD.

## INTRODUCTION

Post-stroke depression (PSD) has been considered the most frequent and important neuropsychiatric consequence of stroke [1]. It was reported that the incidence of PSD was approximately 30% within two weeks, 60% within one year and 80% within two years after stroke attack [2, 3]. Nevertheless, treatment with antidepressant drugs provides little benefit for patients with PSD [4]. It is traditionally believed that stroke directly damages the neural circuits, such as those in the frontal and temporal lobes, that are associated with depression. However, a meta-analysis showed that the relation between the development of PSD and the lesion location was uncertain [5]. Another study reported that no correlation was observed between the lesion side or location, including the frontal or temporal lobe, and the incidence and severity of PSD [6].

In previous years, several neural iconography studies have found that psychiatric disorders, such as depression and autism, were associated with cerebellar lesions [7, 8]. However, reports about the relationship between the cerebellum and PSD are scarce. PSD may have a performance and pathogenesis that are similar to those for other types of depression. And the cerebellum may be involved in the development of PSD [9]. Metabolic changes in the cerebellum were measured using N-acetyl aspartic acid (NAA), choline (Cho), and creatine (Cr). Cho is a component of the cell membrane and is involved in phospholipid metabolism. Cho levels reflect the levels of cell membrane transport functioning and cell proliferation, and it also closely correlates with the precursors of the neurotransmitter acetylcholine and mood disorders. NAA is a marker of neuronal activity, the main source of NAA is neurons and their axons. The cerebellum of patients with autistic disorder was observed to have reduced concentrations of NAA compared with controls [10–13]. Cr is a metabolite of energy metabolism with relatively constant level in brain, and it’s usually used as an internal control since its concentration remains relatively constant under various pathophysiological circumstances [14–16]. The ratios of NAA/Cr, Cho/Cr can reflect the changes in Cho and NAA more objectively compared to the absolute concentrations of NAA and Cho [17–19]. The aim of this study was to explore the metabolic changes in the cerebellum in patients with PSD and to analyze the cerebellar contribution to the development and severity of depression using proton magnetic resonance spectroscopy (^1^H-MRS).

## RESULTS

**Figure 1:**
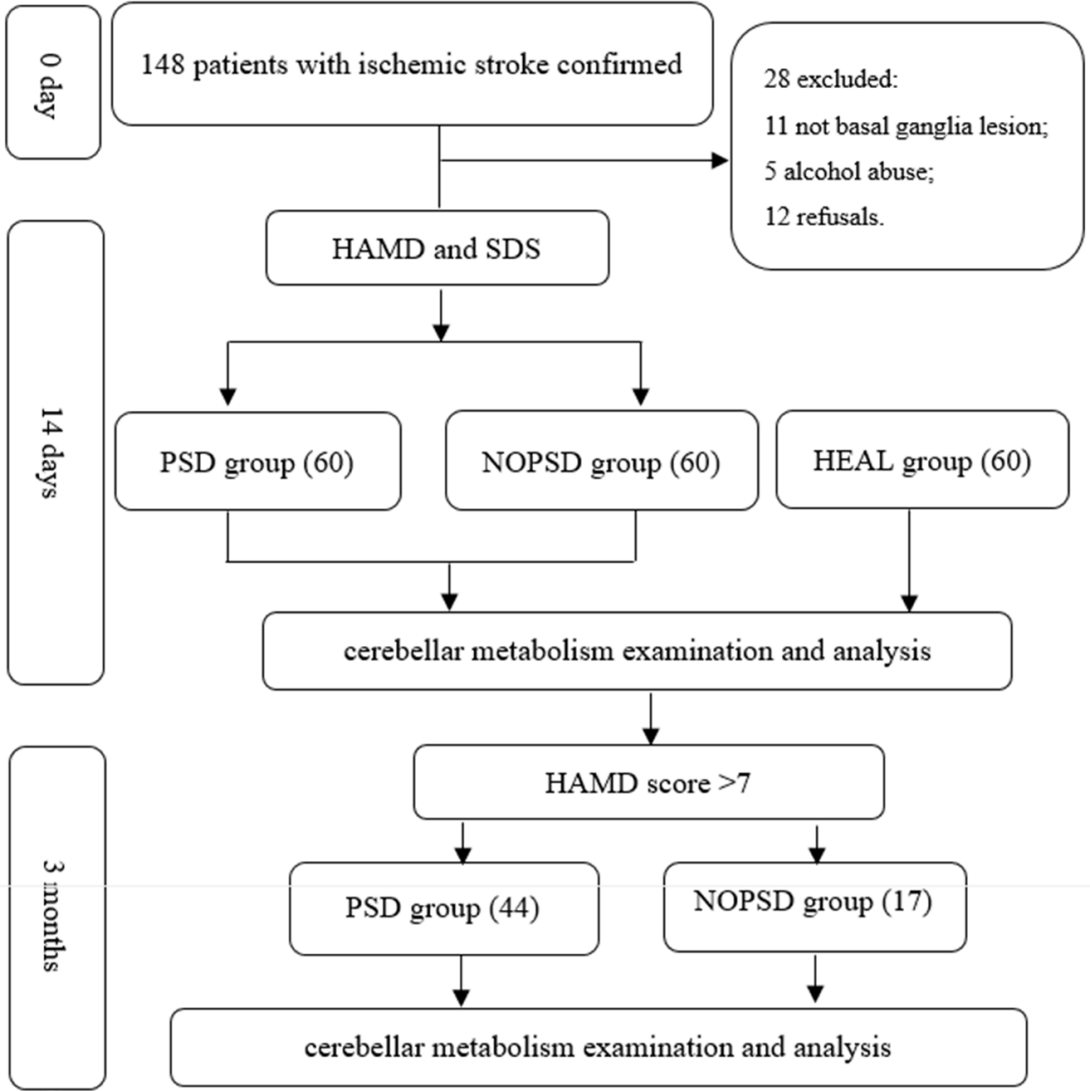
Flow chart of the study design

A total of 148 patients with ischemic stroke confirmed by MRI were selected from the Department of Neurology at the First Affiliated Hospital, Jinzhou Medical University, and 60 healthy volunteers who were each matched by age and education to a patient in the PSD group were included, from August 2014 to April 2016. All patients were included in the study within 7 days of a confirmed ischemic stroke. There were 28 patients were excluded (11 patients with a lesion not involving the basal ganglia, 5 patients with alcohol abuse, 12 refusals). And finally the 3 groups included 60 patients with PSD (PSD group), 60 stroke patients without depression (NOPSD group), and 60 healthy volunteers (HEAL group), see Figure 1.

### Comparison of clinical data

Difference was observed in the NIHSS scores between the PSD and NOPSD groups 14 days after the ischemic stroke occurred (*P* = 0.001). Differences were observed in the physical work (*P* = 0.045), HAMD (*P* < 0.001) and SDS (*P* < 0.001) scores in the PSD group compared to the NOPSD and HEAL groups. The results are shown in Table 1.

**Table 1:** Characteristics of clinical information for PSD, NOPSD and HEAL groups

Variables	PSD (*N* = 60)	NOPSD (*N* = 60)	HEAL (*N* = 60)	Statistics	*P* value
Age (range)	59.40 ± 9.98 (54–72)	55.28 ± 9.54 (47–69)	57.33 ± 10.21 (49–70)	F = 2.591	0.078
Gender (Male/Female)	33/27	29/31	32/28	χ^2^ = 0.579	0.749
Education years	8.99 ± 2.54	9.08 ± 2.61	9.23 ± 2.38	F = 0.140	0.860
Physical work [ N (%)]	31(51.7)	19(31.7)	20(33.3)	χ^2^ = 6.218	0.045^a^
Average income > 1000 yuan [N (%)]	34(56.7)	30(50.0)	35(58.3)	χ^2^ = 0.943	0.624
Healthy insurance [N (%)]	34(56.7)	31(51.7)	35(58.3)	χ^2^ = 0.585	0.746
Hypertension history [N (%)]	32(53.3)	27(45.0)	25(41.7)	χ^2^ = 1.741	0.419
Diabetes history [N (%)]	22(36.7)	19(31.7)	27(45.0)	χ^2^ = 2.316	0.314
Heart disease history [N (%)]	19(31.7)	19(31.7)	13(21.7)	χ^2^ = 1.970	0.373
Hyperlipidemia history [N (%)]	25(41.7)	23(38.3)	23(38.3)	χ^2^ = 0.186	0.911
Hypertension family history [N (%)]	27(45.0)	20(33.3)	22(36.7)	χ^2^ = 1.833	0.400
Heart disease family history [N (%)]	26(43.3)	23(38.3)	24(40.0)	χ^2^ = 0.323	0.851
Stroke family history [N (%)]	25(41.7)	27(45.0)	29(48.3)	χ^2^ = 0.539	0.764
Past lifestyles	1.70 ± 1.11	1.63 ± 1.10	1.73 ± 1.12	F = 0.128	0.882
APGAR scores	6.73 ± 1.82	7.18 ± 1.50	7.28 ± 1.90	F = 1.684	0.188
NIHSS scores (when ischemic stroke occurred)	6.70 ± 2.13	6.22 ± 2.23	-	t = 1.206	0.227
NIHSS scores (14 days after ischemic stroke)	4.97 ± 1.82	3.87 ± 1.57	-	t = 3.545	0.001
HAMD scores	13.20 ± 3.77	2.87 ± 1.84	2.68 ± 1.78	F = 314.080	< 0.001^b^
SDS scores	57.47 ± 3.72	31.03 ± 7.14	29.03 ± 6.65	F = 415.967	< 0.001^c^

a: PSD vs. NOPSD: χ^2^ = 4.937, *P* = 0.026; PSD vs. HEAL: χ^2^ = 4.126, *P* = 0.042; NOPSD vs. HEAL: χ^2^ = 0.038, *P* = 0.845.

b: PSD vs. NOPSD: t = 21.505, *P* < 0.001; PSD vs. HEAL: t = 21.900, *P* < 0.001; NOPSD vs. HEAL: t = 0.396, *P* = 0.693.

c: PSD vs. NOPSD: t = 24.021, *P* < 0.001; PSD vs. HEAL: t = 25.838, *P* < 0.001; NOPSD vs. HEAL: t = 1.817, *P* = 0.071.

### Comparison of iconography data from examination on 14th day after stroke

#### Comparisons of cerebral white matter lesions and cerebral infarction volumes

There were no differences among three groups in the ARWMC scores for the frontal lobe, parietal lobe, occipital lobe, temporal lobe, infratentorial area, or basal ganglia (*P* > 0.05). However, the difference in the total scores for all areas were observed among the three groups (*P* = 0.002). There was no difference in the lesion location and standard brain infarct volume between the PSD group and NOPSD group (*P* > 0.05). The results are shown in Table 2.

**Table 2:** Comparison of cerebral white matter lesion and cerebral infarction volume from examination at 14 days after stroke

Variables	PSD	NOPSD	HEAL	Statistics	*P* value
ARWMC scores					
Frontal lobe	1.517 ± 1.081	1.367 ± 1.041	1.167 ± 1.044	F = 1.661	0.193
Parietal occipital lobe	1.783 ± 1.342	1.483 ± 1.186	1.367 ± 1.089	F = 1.888	0.154
Temporal lobe	0.667 ± 0.752	0.583 ± 0.770	0.517 ± 0.624	F = 0.657	0.503
Infratentorial area	0.717 ± 0.666	0.583 ± 0.646	0.583 ± 0.645	F = 0.844	0.435
Basal ganglia	1.250 ± 1.114	1.183 ± 1.000	1.000 ± 0.803	F = 0.445	0.354
Total Scores	5.933 ± 1.956	5.217 ± 2.116	4.633 ± 1.832	F = 6.545	0.002^a^
Lesions located on the left side [N(%)]	34(56.7)	29(48.3)	-	χ^2^ = 0.835	0.361
Standard brain infarct volume (ml)	4.645 ± 1.842	4.697 ± 2.206	-	t = 0.140	0.889

^a^PSD vs. NOPSD: t = 24.021, *P* < 0.001; PSD vs. HEAL: t = 25.838, *P* < 0.001; NOPSD vs. HEAL: t = 1.817, *P* = 0.071.

#### Comparison of the metabolites detected by MRS

**Figure 2:**
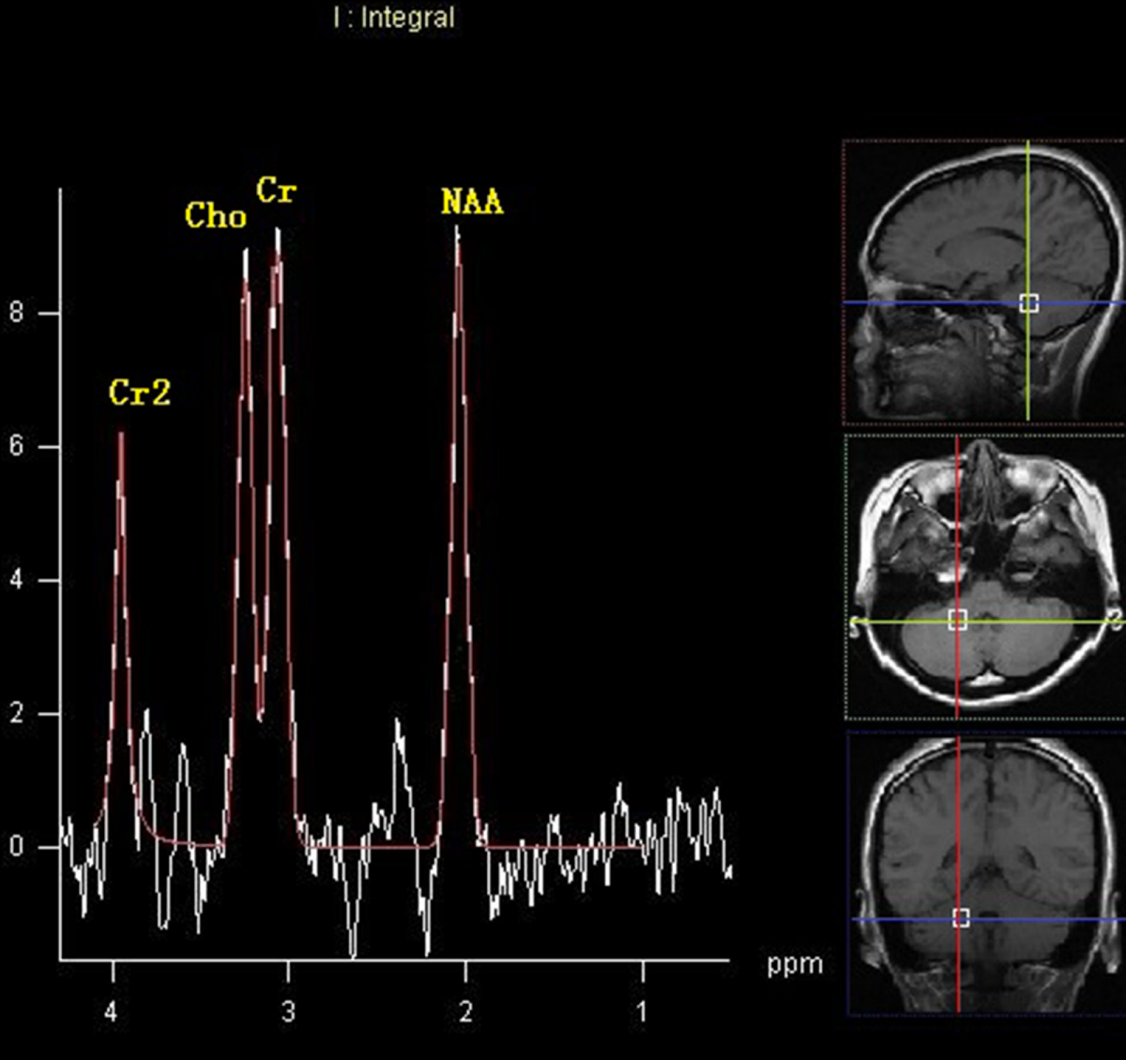
MRS image of cerebellar hemisphere contralateral to the lesion in the patients with PSD at 14 days after stroke

**Figure 3:**
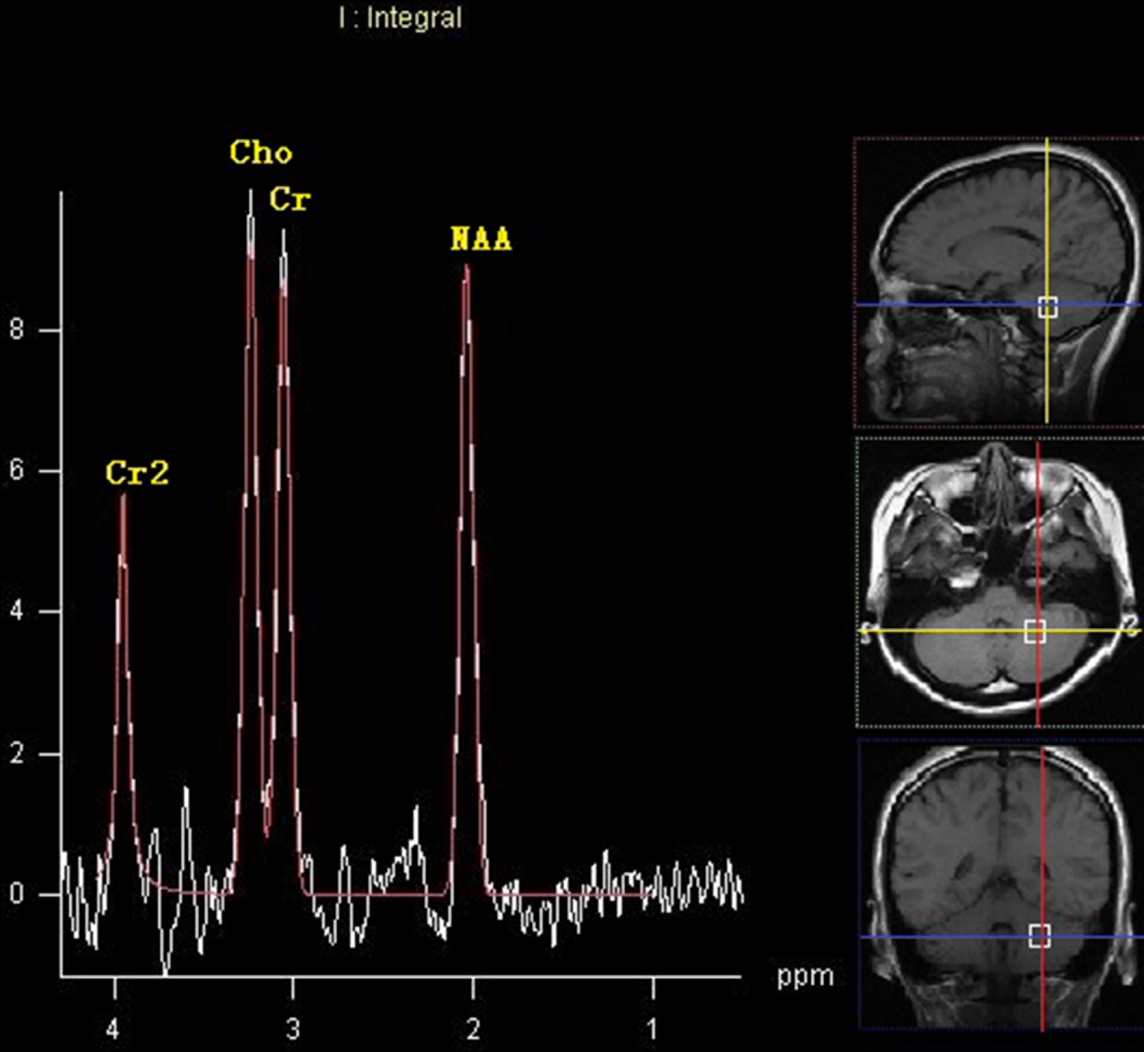
MRS image of cerebellar hemisphere ipsilateral to the lesion in the patients with PSD at 14 days after stroke

No differences were observed in ratios of NAA/Cr, Cho/Cr and Cho/NAA ratios between the left and right cerebellum within the HEAL group (*P* > 0.05). There were no differences in the ratios of NAA/Cr, Cho/Cr and Cho/NAA in either cerebellar hemisphere between the NOPSD group and HEAL group (*P* > 0.05). There were no differences in the NAA/Cr ratios in either cerebellar hemisphere among the three groups (*P* > 0.05), and the Cho/Cr and Cho/NAA ratios in the cerebellar hemisphere ipsilateral to the lesion were similar with those in HEAL and NOPSD groups (*P* > 0.05). The Cho/Cr and Cho/NAA ratios in the cerebellar hemisphere contralateral to the lesion were higher in the PSD group than those in the HEAL and NOPSD groups (*P* < 0.05). Within the PSD group, the Cho/Cr and Cho/NAA ratios in the cerebellar hemisphere contralateral to the stroke lesion were higher than those in the cerebellar hemisphere ipsilateral to the stroke region (*P* < 0.05). The results are shown in Table 3 and Figures 2 and 3.

**Table 3:** Comparison of the metabolites measured by MRS among PSD, NOPSD and HEAL groups at 14 days after stroke

	NAA/Cr				Cho/Cr				Cho/NAA			
	Lesion ipsilateral	Lesion contralateral	t value	*P1* value	Lesion ipsilateral	Lesion contralateral	t value	*P1* value	Lesion ipsilateral	Lesion contralateral	t value	*P1* value
PSD	1.043 ± 0.047	1.041 ± 0.040	0.251	0.797	0.782 ± 0.052	0.863 ± 0.070	7.195	< 0.001	0.751 ± 0.056	0.830 ± 0.075	6.538	< 0.001
NOPSD	1.039 ± 0.070	1.041 ± 0.059	0.169	0.872	0.787 ± 0.074	0.792 ± 0.058	0.412	0.633	0.762 ± 0.105	0.764 ± 0.086	0.114	0.903
HEAL	1.040 ± 0.055	1.043 ± 0.064	0.275	0.729	0.780 ± 0.084	0.784 ± 0.071	0.282	0.762	0.757 ± 0.093	0.751 ± 0.085	0.369	0.680
F value	0.086	0.039	-	-	0.128	10.344	-	-	0.250	6.981	-	-
*P* value	0.917	0.962	-	-	0.880	< 0.001^a^	-	-	0.779	< 0.001^b^	-	-

a: PSD vs. NOPSD: t = 5.839, *P* < 0.001; PSD vs. HEAL: t = 6.497, *P* < 0.001; NOPSD vs. HEAL: t = 0.658, *P* = 0.511.

b: PSD vs. NOPSD: t = 4.401, *P* < 0.001; PSD vs. HEAL: t = 5.267, *P* < 0.001; NOPSD vs. HEAL: t = 0.866, *P* = 0.387.

#### Multiple linear regression analysis for factors related to PSD

As shown in Tables 4 and 5, the HAMD score was a dependent variable. The Cho/Cr ratio in the cerebellar hemisphere contralateral to the lesion showed a positive relationship with the HAMD scores (R = 0.358, *P* < 0.05). Likewise, the Cho/NAA ratio in the cerebellar hemisphere contralateral to the lesion showed a positive relationship with the HAMD scales (R = 0.294, *P* < 0.05).

**Table 4:** Multiple linear regression analysis for factors related to PSD at 14 days after stroke

Variables	Non-Standardization		Standardization Partial regression coefficients	t value	*P* value	95% confidence interval	
	Partial regression coefficients	Standard error				Lower	Upper
Physical work	–0.660	0.945	–0.088	–0.698	0.488	–2.555	1.234
NIHSS score at 14th day	–0.284	0.269	–0.137	–1.053	0.297	–0.823	0.256
ARWMC total scores	0.089	0.248	0.046	0.359	0.721	–0.409	0.587
Cho/Cr ratio	18.577	7.007	0.346	2.651	0.010	4.534	32.619

**Table 5:** Multiple linear regression analysis for factors related to PSD at 14 days after stroke

Variables	Non-Standardization		Standardization Partial regression coefficients	t value	*P* value	95% confidence interval	
	Partial regression coefficients	Standard error				Lower	Upper
Physical work	–0.383	0.992	–0.051	–0.387	0.701	–2.370	1.604
NIHSS score at 14th day	–0.256	0.276	–0.124	–0.927	0.358	–0.810	0.298
ARWMC total scores	0.055	0.253	0.029	0.219	0.828	–0.453	0.563
Cho/NAA ratio	13.930	6.829	0.278	2.040	0.046	0.244	27.617

### Comparison of iconography data from examination at 3 months after stroke

The patients in the PSD group and NOPSD group were followed up 3 months. After 3 months, there were 44 patients (16 withdraws) with HAMD score > 7 and average HAMD score 14.41 ± 2.99 in PSD group. There were 17 patients with HAMD score > 7 and average HAMD score 11.06 ± 3.61 in NOPSD group, see Figure 1.

### Comparison of the metabolites detected by MRS

**Figure 4:**
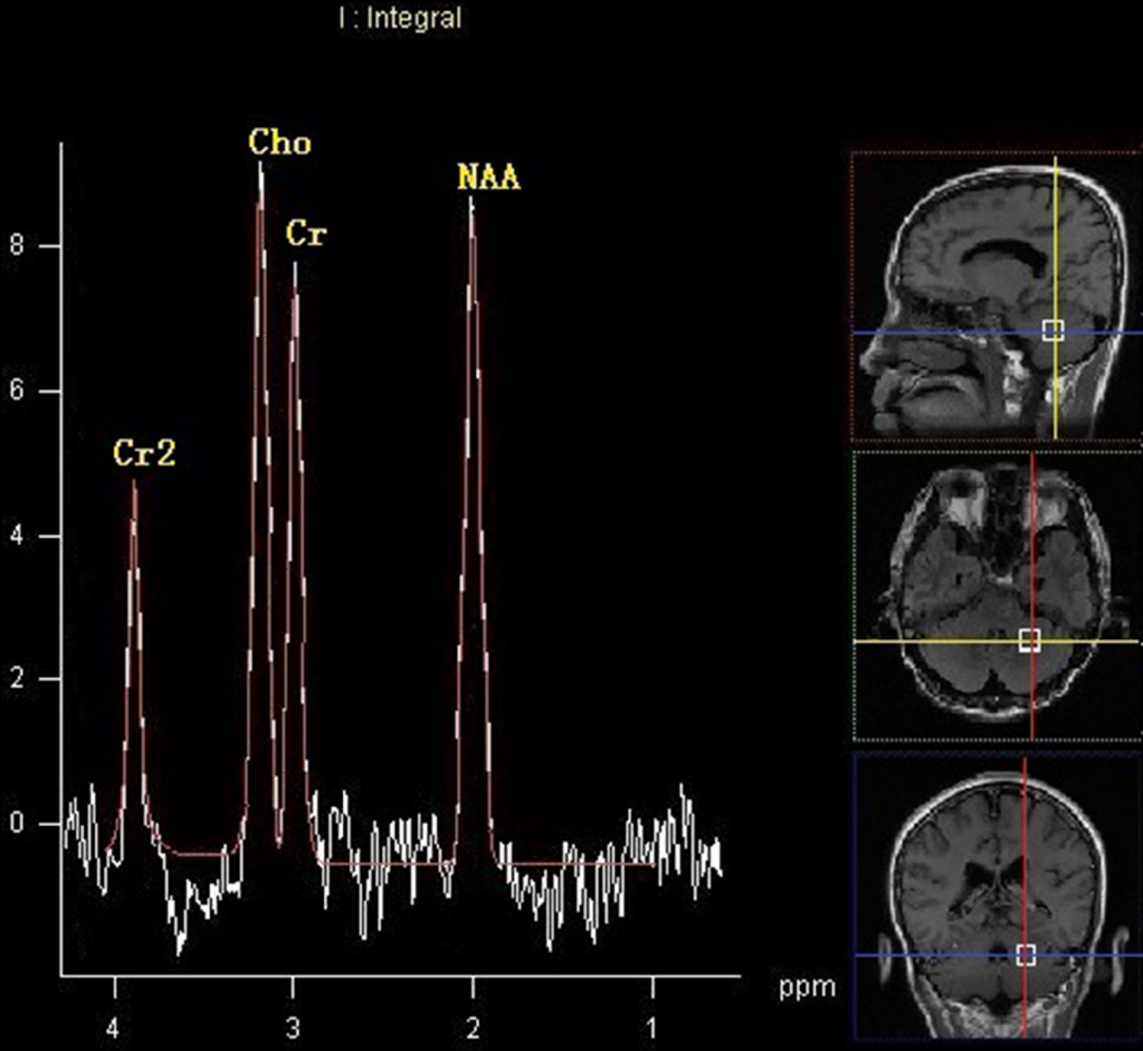
MRS image of cerebellar hemisphere contralateral to the lesion in the patients with PSD at 3 months after stroke

**Figure 5:**
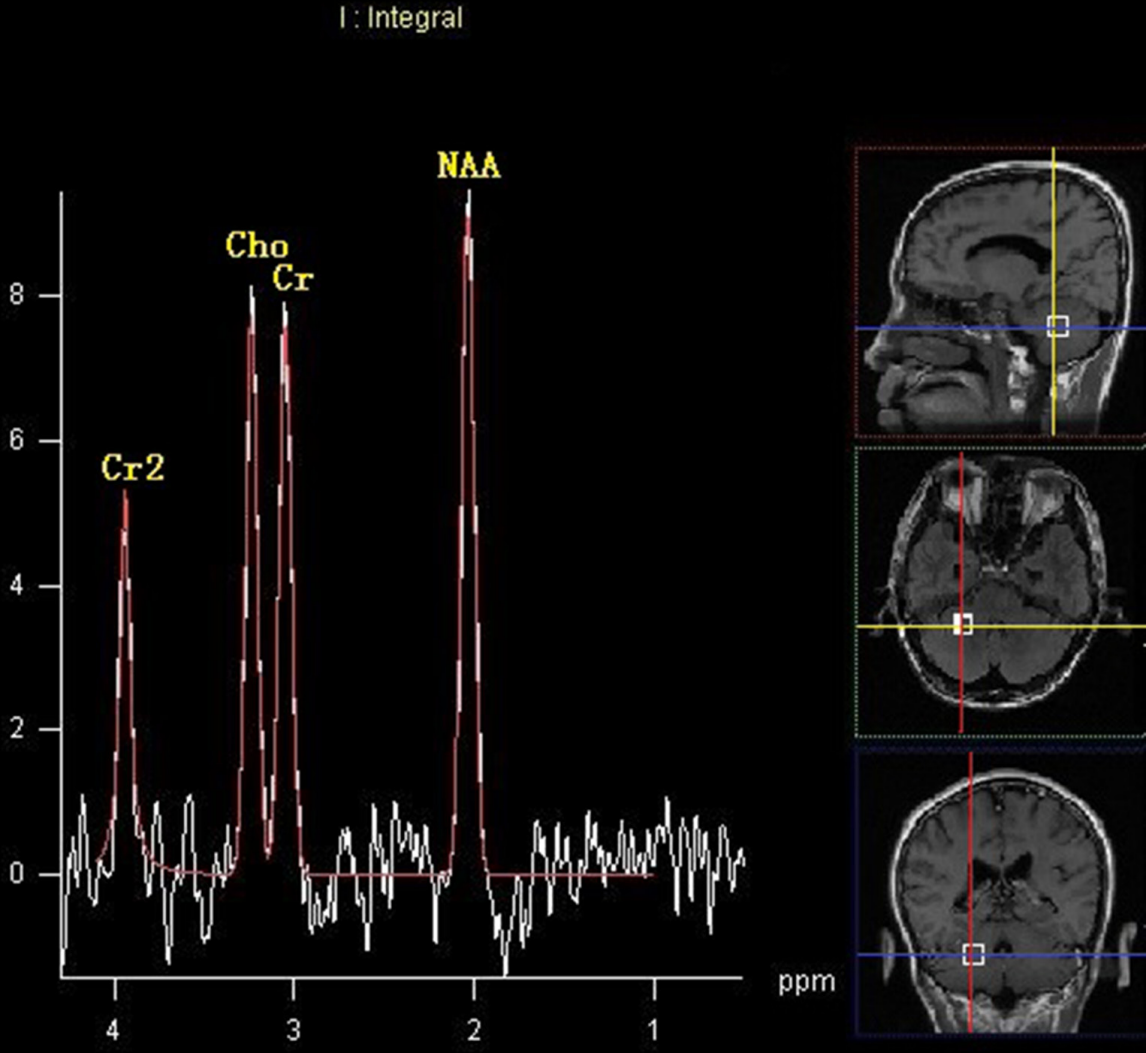
MRS image of cerebellar hemisphere ipsilateral to the lesion in the patients with PSD at 3 months after stroke

For the PSD group, the NAA/Cr ratio in the cerebellar hemisphere contralateral to the lesion at 3 months after stroke was lower than that detected on 14th day after stroke (*P* = 0.041), and lower than that in the cerebellar hemisphere ipsilateral to the lesion at 3 months after stroke (*P* = 0.046).The Cho/Cr, Cho/NAA ratios in the cerebellar hemisphere contralateral to the lesion at 3 months after stroke was higher than that detected on 14th day after stroke (*P* = 0.017, *P* = 0.002), and higher than that in the cerebellar hemisphere ipsilateral to the lesion at 3 months after stroke (*P* < 0.001); No difference was observed in the ratios of NAA/Cr, Cho/Cr, Cho/NAA in the cerebellar hemisphere ipsilateral to the lesion compared to those detected on 14th day after stroke (*P* = 0.865, *P* = 0.230, *P* = 0.242), see Table 6 and Figures 4 and 5.

**Table 6:** Comparison of the metabolites at 14 days, 3 months after stroke in PSD group

	NAA/Cr				Cho/Cr				Cho/NAA			
	Lesion ipsilateral	Lesion contralateral	t value	*P1* value	Lesion ipsilateral	Lesion contralateral	t value	*P1* value	Lesion ipsilateral	Lesion contralateral	t value	*P1* value
14 days after ischemic stroke	1.043 ± 0.047	1.041 ± 0.040	0.251	0.797	0.782 ± 0.052	0.863 ± 0.070	7.195	< 0.001	0.751 ± 0.056	0.830 ± 0.075	6.538	< 0.001
3 months after ischemic stroke	1.042 ± 0.046	1.020 ± 0.638	0.366	0.046	0.796 ± 0.066	0.901 ± 0.090	7.287	< 0.001	0.766 ± 0.074	0.887 ± 0.107	7.204	< 0.001
t value	0.171	2.073	-	-	1.208	2.437	-	-	1.178	3.219	-	-
*P* value	0.865	0.041	-	-	0.230	0.017	-	-	0.242	0.002	-	-

For the NOPSD group, the Cho/Cr ratio in the cerebellar hemisphere contralateral to the lesion was higher than that in the cerebellar hemisphere ipsilateral to the lesion (*P* = 0.037), and higher than that in the cerebellar hemisphere contralateral to the lesion detected on 14th day after stroke (*P* = 0.045); No difference was observed in the ratios of NAA/Cr, Cho/Cr, Cho/NAA in the cerebellar hemisphere ipsilateral to the lesion compared to those detected on 14th day after stroke (*P* > 0.05), see Table 7.

**Table 7:** Comparison of the metabolites at 14 days, 3 months after stroke in NOPSD group

	NAA/Cr				Cho/Cr				Cho/NAA			
	Lesion ipsilateral	Lesion contralateral	*t* value	*P1* value	Lesion ipsilateral	Lesion contralateral	*t* value	*P1* value	Lesion ipsilateral	Lesion contralateral	*t* value	*P1* value
14 days after ischemic stroke	1.039 ± 0.070	1.041 ± 0.059	0.169	0.872	0.787 ± 0.074	0.792 ± 0.058	0.412	0.633	0.762 ± 0.105	0.764 ± 0.086	0.114	0.903
3 months after ischemic stroke	1.044 ± 0.062	1.043 ± 0.054	0.094	0.944	0.776 ± 0.058	0.824 ± 0.056	3.612	0.037	0.746 ± 0.068	0.793 ± 0.076	2.570	0.093
t value	0.268	0.133	-	-	0.524	2.041	-	-	0.607	1.240	-	-
*P* value	0.790	0.894	-	-	0.602	0.045	-	-	0.546	0.219	-	-

### Correlation between metabolites and HAMD scores

In the PSD group, negative correlation between NAA/Cr ratio and HAMD (*P* = 0.030), positive correlation between Cho/Cr (*P* = 0.002), Cho/NAA (*P* < 0.001) ratios and HAMD were observed in the cerebellar hemisphere contralateral to the lesion, while no correlation between NAA/Cr, Cho/Cr, Cho/NAA ratios and HAMD were observed in the cerebellar ipsilateral contralateral to the lesion, see Table 8.

**Table 8:** Correlation of the metabolites and HAMD scores at 3 months after stroke in PSD group

	NAA/Cr		Cho/Cr		Cho/NAA	
	Lesion ipsilateral	Lesion contralateral	Lesion ipsilateral	Lesion contralateral	Lesion ipsilateral	Lesion contralateral
R value	0.152	–0.328	0.244	0.460	0.191	0.562
*P* value	0.324	0.030	0.110	0.002	0.214	< 0.001

In the NOPSD group, positive correlation between Cho/Cr ratio (*P* = 0.033) and HAMD were observed in the cerebellar hemisphere contralateral to the lesion, while no correlation between NAA/Cr, Cho/Cr, Cho/NAA ratios and HAMD were observed in the cerebellar ipsilateral contralateral to the lesion, see Table 9.

**Table 9:** Correlation of the metabolites and HAMD scores at 3 months after stroke in NOPSD group

	NAA/Cr		Cho/Cr		Cho/NAA	
	Lesion ipsilateral	Lesion contralateral	Lesion ipsilateral	Lesion contralateral	Lesion ipsilateral	Lesion contralateral
R value	0.297	0.129	0.338	0.518	0.091	0.278
*P* value	0.247	0.645	0.185	0.033	0.728	0.280

## DISCUSSION

Changes in the local cerebral blood supply and metabolism due to neurophysiological activity can lead to changes in the ^1^H-MRS signal [20]. Therefore, ^1^H-MRS is a useful tool for the early identification of PSD and to achieve early intervention, thus improving the physical and cognitive functions in patients with PSD [21, 22]. The pathogenesis of PSD, however, remains unclear. Our preliminary studies have demonstrated changes in the structure and function of the cerebellum in rats with PSD compared to the healthy rats [9, 23]. However, studies on the correlation between changes in cerebellar metabolism and the development and severity PSD in patients were still scarce. Lassalle-Lagadec found emotional cognition after stroke may be related to cerebellar volume [24]. And two reports have found possible neural mechanisms for the involvement of the cerebellum in patients with depression by fMRI examination [25, 26]. Peng showed abnormalities of cortical-limbic-cerebellar white matter networks may lead to treatment-resistant depression [7]. Therefore, cerebellum may play an important role in development of PSD. Especially, damage of the basal ganglia may produce a higher frequency of depressive disorders since these structures are the most important subcortical/cortical gateway. Most of the neuronal pathways implicated in mood have to transit the basal ganglia and surrounding white matter. And more than 80% of the old patients with ischemic stroke due to unilateral basal ganglia lesions can lead to PSD in China [2, 15].

On the 14th day after stroke, the Cho/Cr, Cho/NAA ratios in the cerebellar hemisphere contralateral to the lesion in the PSD group was higher than those for the HEAL and NOPSD groups; Further, in the PSD group, the Cho/Cr, Cho/NAA ratios in the cerebellar hemisphere contralateral to the lesion was much higher at 3 months after stroke compared to that at 14 days after stroke, indicating the cerebellar metabolism in patients with PSD had changed, and the changes may be more significant with time. The increasing Cho/Cr ratios suggested that PSD may affect membrane phospholipids in neurons during periods of increased cerebellar tissue renewal, when changes in the membrane structure occur. In autism studies, the cerebellar Cho concentration was higher in patients than those in the normal control group [8, 10], which is similar to our present findings. The Xu et al. study showed the Cho concentration in patients with PSD was not higher compared to the normal control group. However, this discrepancy may be explained by methodological differences between studies, including the study subjects, ROI locations, and image resolution [27].

Contrarily, we found no differences in the Cho/Cr, NAA/Cr and Cho/NAA ratios in either cerebellar hemisphere between the NOPSD group and HEAL group, which suggests cerebellar metabolism of patients without PSD may not change, or the cerebellar metabolism in patients without PSD was similar to that in healthy people; However, at 3 months after stroke, the results from the patients developed PSD in NOPSD group showed that, the Cho/Cr ratio in the cerebellar hemisphere contralateral to the lesion was higher than that detected on 14th day after stroke. These results further support that cerebellar metabolism may be correlated with PSD.

Crossed cerebellar diaschisis (CCD) is the interruption of fiber tracts targeting the cerebellar hemispheres, namely the corticopontocerebellar (CPC) tract, that can result from a variety of brain injuries. CCD prevents cortical excitatory impulses from transmitting through the pons and medipeduncle to the contralateral cerebellar hemisphere, leading to a decreased blood flow, metabolism decline, and functional inhibition in that cerebellar hemisphere [28, 29]. Strakowski et al. suggested that the reticular formation and the limbic system are functional centers related to affective disorders [30]. There are extensive fiber tracts between the cerebellum and these systems, and one of the most important fiber tracts is the CPC tract [31, 32]. In this study, a lesion in the basal ganglia interrupted this pathway and resulted in CCD, and this affected the metabolism of the contralateral cerebellum and may have led to PSD.

The cerebellar NAA/Cr ratios were also measured in patients with PSD. No difference was observed in the NAA/Cr ratios for the left or right cerebellar hemisphere between the PSD group and control group. NAA is related to the integrity of neurons and reflects their functional state. This result may suggest that the pathogenesis of acute PSD was related to non-neuronal changes. Other studies also showed no differences in cerebellar NAA concentrations in PSD patients compared to a control group [33, 34]. However, lower NAA/Cr ratio in the cerebellar hemisphere contralateral to the lesion at 3 months after stroke compared to that at 14 days after stroke indicated that, the pathogenesis of chronic PSD may be related to the abnormal function of neurons and axons, abnormal cell energy metabolism and structural damage.

Several studies using the functional magnetic resonance imaging (fMRI) technique revealed changes in cerebellar functioning in patients with depression [25, 26]. Other studies also found changes in the cerebellar structure of patients with PSD, and such structural changes will inevitably lead to changes in function [7, 24]. A magnetic resonance diffusion tensor imaging study on depression also found cerebellar white matter abnormalities in patients with PSD, suggesting the cerebellar structure had changed [9]. Similarly, studies have confirmed that electrical stimulation of the cerebellum can improve symptoms in patients with PSD [13, 35].

The limitations of this study were the relatively small sample size in NOPSD group at 3 months after stroke and no detection of other metabolites (such as inositol, glutamate and other small molecules), which may provide additional valuable information.

### Subjects and methods

#### Subjects

#### PSD group

The inclusion criteria were as follows: (1) patients who were first diagnosed with a unilateral basal ganglia ischemic stroke confirmed by MRI and according to the guidelines for acute ischemic stroke diagnosis released by the Chinese Society of Neurology in 2014 [14, 15]; (2) depression that was diagnosed on the 14th day after the ischemic stroke consistent with the diagnostic criteria for depression from the Chinese Classification and Diagnostic Criteria of Mental Disorders-Third Edition (CCMD-3) with a score for the Self-Rating Depression Scale (SDS) ≥ 53 points and a score for the 17-item Hamilton Depression Scale (HAMD) > 7 points [14, 16]; (3) patients had received care from their family members before and 1 weeks after the ischemic attack. The exclusion criteria were as follows: (1) age > 80 years; (2) history of cerebrovascular disease (cerebral infarction or cerebral hemorrhage), traumatic brain injury or organic brain disease; (3) a lesion not involving the basal ganglia; (4) mental retardation, severe visual or hearing impairment, or aphasia; (5) personal or family history of psychiatric diseases before diagnosed with the ischemic stroke, including depression and anxiety; (6) suspected history of alcohol or drug abuse; (7) use of mannitol and steroids in the treatment; (8) coexist with life stress (refers to the serious negative life events, such as serious illness, loss of important relationship, and so on).

#### NOPSD group

Depression was evaluated on the 14th day after ischemic stroke attack, and patients with depression were excluded according to the CCMD-3 and the scores for the SDS (< 53 points) and HAMD (≤ 7 points). All other inclusion and exclusion criteria were the same as those for the PSD group.

#### HEAL group

All subjects had no personal or family history of depression, anxiety, traumatic brain injury, or organic brain disease. They were all right-handed, and depression was excluded using the SDS and HAMD.

The study was carried out according to the Declaration of Helsinki and Good Clinical Practice guidelines. This study was approved by the Medical Ethics Committee of the First Affiliated Hospital of Jinzhou Medical University, and each participant signed an informed consent form. All methods were conducted in accordance with the approved guidelines.

### Collection of clinical information

Age, gender, family function, educational degree, occupation, family economic status, health insurance, personal and family history (hypertension, diabetes, hyperlipidemia, heart diseases, stroke), and lifestyle characteristics (smoking, drinking, physical exercise and intake of vegetables and fruits) were recorded for all study participants in their initial visits. The information was provided by the patients or their family members. Brain CT or MRI examination, echocardiography, electrocardiography, carotid ultrasonography, and TCD were also performed.

The patient’s career was categorized as physical or mental work. The family economic status was either low or high according to the monthly income (low: ≤ 1000 yuan, versus high: > 1000 yuan). Medical insurance was categorized as either having medical insurance or having no medical insurance [17]. Questions regarding the personal and family history were answered as “Yes” or “No”. Hypertension, diabetes, and dyslipidemia were categorized as diseases that were diagnosed previously or during this treatment period. An SBP ≥ 140 mmHg or DBP ≥ 90 mmHg was diagnosed as hypertension; an FPG ≥ 11.1 mmol/L was diagnosed as diabetes; a total cholesterol (TC) > 5.6 mmol/L, triglyceride (TG) > 1.7 mmol/L, low-density lipoprotein cholesterol (LDL-C) > 3.4 mmol/L, or high-density lipoprotein cholesterol < 0.9 mmol/L (HDL-C) was diagnosed as dyslipidemia. Coronary heart disease, atrial fibrillation, or cardiac valve disease that was diagnosed or a pacemaker implantation or cardiac surgery that occurred either before the study or during this treatment period was considered a history of heart disease [18]. The lifestyle was defined as the daily behavior before the stroke attack. It has been proven that smoking, drinking, physical exercise, and consumption of vegetables and fruits are related to the occurrence and prevention of stroke attack. Each lifestyle characteristic was categorized as a healthy or an unhealthy style. If a characteristic was unhealthy, then the number value 1 was assigned; otherwise, the number value 0 was assigned. The number of unhealthy characteristics ranged from 0 to 4. In addition, smoking was defined as at least 1 cigarette per day for more than 6 months. Drinking was defined as a drink containing more than 50 ml of liquors, 100 ml of wine (rice wine) or 300 ml of beer per day. A lack of physical exercise was defined as an average physical activity time that was less than 0.5 hour per day, whereas a lack of vegetable and fruit consumption was defined as an intake that was less than 450 g per day [2, 15].

## MATERIALS AND METHODS

### Study tools

All tests were performed by a double-blinded, experienced, trained neurologist. (1) The Family APGAR index was administered to assess a family member’s perception of family functioning by examining his or her satisfaction with family relationships. The scores for the APGAR index range from 0 to 10. Scores of 7–10 represent a favorable family function, scores of 4–6 represent a moderate family dysfunction, and scores of 0–3 represent a severe family dysfunction. (2) The extent of neurological impairment after stroke was evaluated with the National Institutes of Health Stroke Scale (NIHSS). The NIHSS was administered when the ischemic stroke occurred and on day 14 after the stroke. The scores for the NIHSS range from 0–42, and higher scores indicate a more severe stroke. (3) Depression was evaluated 14 days and 3 months after ischemic stroke, respectively. The SDS was used to screen for depression, whereas the HAMD was used to measure the severity of depression. (4) White matter lesions were assessed 14 days after ischemic stroke. The Age-Related White Matter Changes scale (ARWMC) is widely used to evaluate the location and severity of white matter lesions [19]. In this study, we used the revised version of this scale from Xiong et al. [20]. In the ARWMC, the cerebral hemispheres are subdivided into five areas, including the frontal lobe, occipital lobe, temporal lobe, infratentorial area (including the cerebellum and brain stem), and basal ganglia, and each area is assessed separately (score 0–3; 4 levels). The final score is the sum of all points for both cerebral hemispheres combined (0 to 30 points).

### MRI brain morphology analysis

3T high-field magnetic resonance (GE Medical Systems) was used with a circularly polarized head coil. Compared with 1.5T MRI, it can provide improvements in both signal/noise ratio and contrast/noise ratio, and can be used to improve image homogeneity, spatial and temporal resolution. Brain MRI morphological information was collected 14 days and 3 months after ischemic stroke using T1 WI, T2 WI and DWI of high-field strength MRI sequences, respectively. A semi-automatic method was used to measure the infarcts. The DWI sequence was used to find the responsible lesions, and the contours of the infarct in each layer of the DWI sequence were manually sketched. The infarct areas were then calculated and multiplied by the layer spacing, and the sum of all infarction volumes was the volume of cerebral infarction. Finally, the volume of the cerebral infarction was standardized and processed using a picture archiving and communication system (PACS, Huahai Medicine) and according to the following formula: standard brain infarct volume= infarct volume×average brain median sagittal plane area/median sagittal plane area [21]. The scanning parameters for axial T1 WI RTSE were as follows: TR = 2000 ms, TE = 20 ms, IR = 800 ms, section thickness 6 mm, interval 1 mm, NSA = 1, matrix = 400 × 351, TSE Factor = 7; T2 WI DRIVE: TR = 3000 ms, TE = 80 ms, section thickness 6 mm, interval 1 mm, NSA = 1, matrix = 484 × 377, TSE factor, 15. The scanning parameters for FLAIR sequences were as follows: TR = 11000 ms, TE = 120 ms, IR = 2800 ms, section thickness 6 mm, interval 1 mm, NSA = 1, matrix = 240 × 187, TSE Factor = 32; sagittal T2 W TSE:TR = 2123 ms, TE = 80 ms, section thickness 5 mm, interval 1 mm, NSA = 1, matrix = 384 × 336, TSE Factor = 19.

### Metabolic changes in the cerebellum measured by ^1^H-MRS

^1^H-MRS was performed at the 14 days and 3 months after stroke, respectively. A TSE sequence was performed to exclude brain lesions. The left and right cerebellar peduncles of all subjects were symmetrically selected as the spectral region of interest (ROI) using T1 WI or T2 WI MRI images. Cerebrospinal fluid spaces were excluded to avoid contamination while selecting ROI. And the point resolved spin echo spectroscopy (PRESS) in PROBE-SV package were applied in MRS scanning. The average voxel size was 1 cm × 1 cm × 1.5 cm. All subjects underwent MRS examination with the following scanning parameters: TR = 1500 ms, TE = 35 ms. Data were then analyzed by the Spectral Analysis General Electric/Interactive Data Language program (SAGE/IDL, GE Medical Systems). After spectral offset according to the water peak, data processing was as follows: by multiplying a 3–5 Hz Gaussian line broadening, apodization corrected time-domain signal was used to enhance the signal-to-noise ratio, zero filling, highpass convolution filtering with 20 Hz bandwidth, fast Fourier transform, and zero-order and first-order phase correction. Using line-fitting program of the SAGE/IDL, the peaks at 3.22 ppm, 2.02 ppm, and 3.02 ppm were corresponded to Cho, NAA, Cr, respectively.

All operations were conducted by the same experienced physician who was blind to the group assignments, clinical data, and MR imaging. All images were measured at the same time and recorded to disc.

### Statistical analysis

The mean and standard deviation $\left(\bar{x}\pm s\right)$ were used to describe the quantitative data. SPSS 13.0 was used to conduct the statistical analysis. A chi-square test was used to analyze the qualitative data, and one-way analysis of variance (ANOVA) was used to analyze the quantitative data. Comparisons between means for 2 independent samples were conducted with *t*-tests. Comparisons between any 2 means from 3 groups were conducted with LSD-t tests. Multiple linear regression analysis was used to analyze variables that were significant in the one-way ANOVA at 14 days after stroke. Pearson correlation method was used to analyze the relation between cerebellar metabolism changes and HAMD scale scores at 3 months after stroke. *P* value less than 0.05 was considered to have statistical significance, and all tests were two-sided.

## CONCLUSIONS

In summary, cerebellar metabolism changes in terms of Cho/Cr, Cho/NAA, and NAA/Cr ratios may be correlated with the development of PSD. In the cerebellar hemisphere contralateral to the lesion, the Cho/Cr and Cho/NAA ratios were positively correlated with HAMD scores. Higher Cho/Cr and Cho/NAA ratios, lower NAA/Cr ratio in the cerebellar hemisphere contralateral to the lesion was observed at 3 months after stroke compared to that on 14th day after stroke. Cerebellar damage may lead to PSD, and the degree of cerebellar damage may be associated with severity of PSD.

## Abbreviations

PSD: post-stroke depression
NAA: N-acetyl aspartic acid
Cho: choline
Cr: creatine
^1^H-MRS: proton magnetic resonance spectroscopy
CCD: crossed cerebellar diaschisis
TC: total cholesterol
TG: triglyceride
LDL-C: low-density lipoprotein cholesterol
HDL-C: high-density lipoprotein cholesterol
ARWMC: Age-Related White Matter Changes scale
